# Severe cervical deformity due to neurofibromatosis type 1 treated by posterior spinal fusion using a multi-rod construct: a case report

**DOI:** 10.1007/s43390-026-01430-x

**Published:** 2026-05-20

**Authors:** Shota Shimizu, Masashi Uehara, Shota Ikegami, Yoshinari Miyaoka, Hiroki Oba, Daisuke Kurogochi, Takuma Fukuzawa, Shinji Sasao, Jun Takahashi

**Affiliations:** Department of Orthopaedic Surgery, Shinshu University School of Medicine, 3-1-1 Asahi, Matsumoto, Nagano 390-8621 Japan

**Keywords:** Cervical deformity, Neurofibromatosis type 1, Cervical spondylotic myelopathy

## Abstract

**Purpose:**

Neurofibromatosis type 1 (NF-1) has been associated with scoliosis, cervical deformity, and other spinal deformities. In progressive cases of NF-1-associated cervical deformity, surgical intervention may be required to prevent further deterioration. However, there remain no generally recognized guidelines on the surgical strategy for NF-1-related cervical deformity.

**Case presentation:**

A 39-year-old man with diagnosed NF-1 was airlifted to our hospital due to neck pain and bilateral upper extremity paralysis. X-rays and computed tomography (CT) revealed severe neck deformity, including anterior displacement of the C5 vertebral body. We performed laminectomy (C4-C6), posterior fixation (C2-T2), and autologous bone grafting using a pedicle screw construct with cobalt-chromium rods in addition to accessory rods for reinforcement. No surgery-related adverse events were recorded. Successful bone fusion was confirmed by X-rays and CT 2 years postoperatively, with no noticeable correction loss.

**Conclusion:**

In the reported case of severe cervical deformity attributed to NF-1, posterior spinal fusion using a multi-rod construct achieved favorable outcomes and may be considered a viable treatment option.

## Introduction

Neurofibromatosis type 1 (NF-1) is a genetic disorder caused by a mutation in the *NF1* gene. An estimated 10–77% of patients with NF-1 experience such spinal deformities as scoliosis, cervical deformity, kyphoscoliosis, lordoscoliosis, and spondylolisthesis [1, 2]. In progressive cases of NF-1-associated cervical deformity, surgical intervention is often required to prevent further deterioration [3, 4]. However, no generally recognized guidelines on surgical intervention strategies for NF-1-related cervical deformity exist to date, with single posterior instrumented fusion exhibiting high rates of pseudoarthrosis and correction loss (63%) [3, 5].

We herein report a case in which posterior spinal fusion using a multi-rod construct yielded favorable outcomes for severe cervical deformity attributed to NF-1.

## Case presentation

A 39-year-old man with NF-1 was airlifted to our hospital due to neck pain and bilateral upper extremity paralysis. He presented with paresthesia extending from both wrist joints to the fingertips. Manual muscle testing (0–5 scale) revealed the following scores: 0 (right) and 2 (left) for deltoid, 0 (right) and 5 (left) for biceps, 4 (right) and 5 (left) for triceps, 2 (right) and 4 (left) for wrist extension, 2 (right) and 3 (left) for wrist flexion, 2 (right) and 4 (left) for finger flexion, 2 (right) and 4 (left) for finger extension, and 2 (right) and 3 (left) for finger abduction. Lower extremity strength scores were 5 bilaterally for all muscle groups. Pre-existing weakness was noted in the right upper limb, while new weakness was identified in the left upper limb. His Japanese Orthopaedic Association (JOA) score was 7.5 points.

**Fig. 1:**
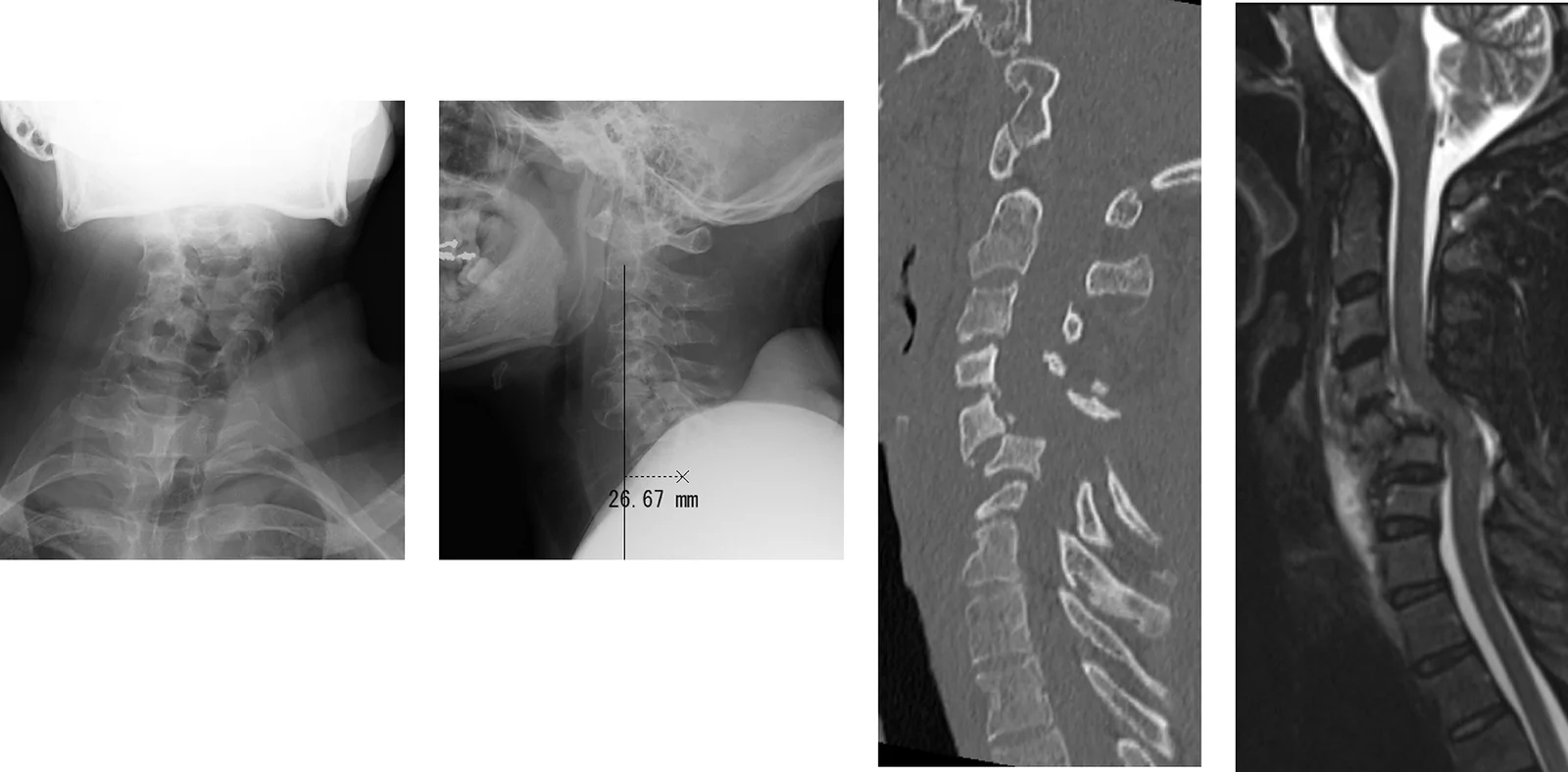
Preoperative X-rays and CT revealed severe neck deformity, including anterior displacement of the C5 vertebral body. Preoperative MRI showed high signal changes in the spinal cord at the C3-C7 levels

X-rays and computed tomography (CT) revealed severe neck deformity, including anterior displacement of the C5 vertebral body (Fig. 1). Magnetic resonance imaging (MRI) showed high signal changes in the spinal cord at the C3-C7 levels (Fig. 1). He exhibited cervical lordosis (CL) of 7° and C2-7 sagittal vertical axis (CSVA) of 26 mm. Preoperative halo-vest traction was attempted to achieve gradual correction. However, minimal radiographic change indicated limited flexibility of the deformity.

Table 1 presents our simplified decision-making algorithm for NF-1-associated cervical deformity. The present case involved multiple tumorous lesions around the cervical spine, creating a condition prone to hemorrhage due to tumor damage. Therefore, we proceeded with a posterior-only approach, with planned second-stage anterior surgery if the initial correction proved insufficient.

**Table 1:** Proposed surgical decision-making algorithm for NF-1-associated cervical deformity

Deformity severity	Anterior column condition	Recommended approach
Mild to moderate	Preserved	Anterior-only or posterior-only
Severe	Preserved	Rigid posterior-only or combined anterior–posterior reconstruction
Severe	Destroyed or osseous fusion	Combined anterior–posterior reconstruction

**Fig. 2:**
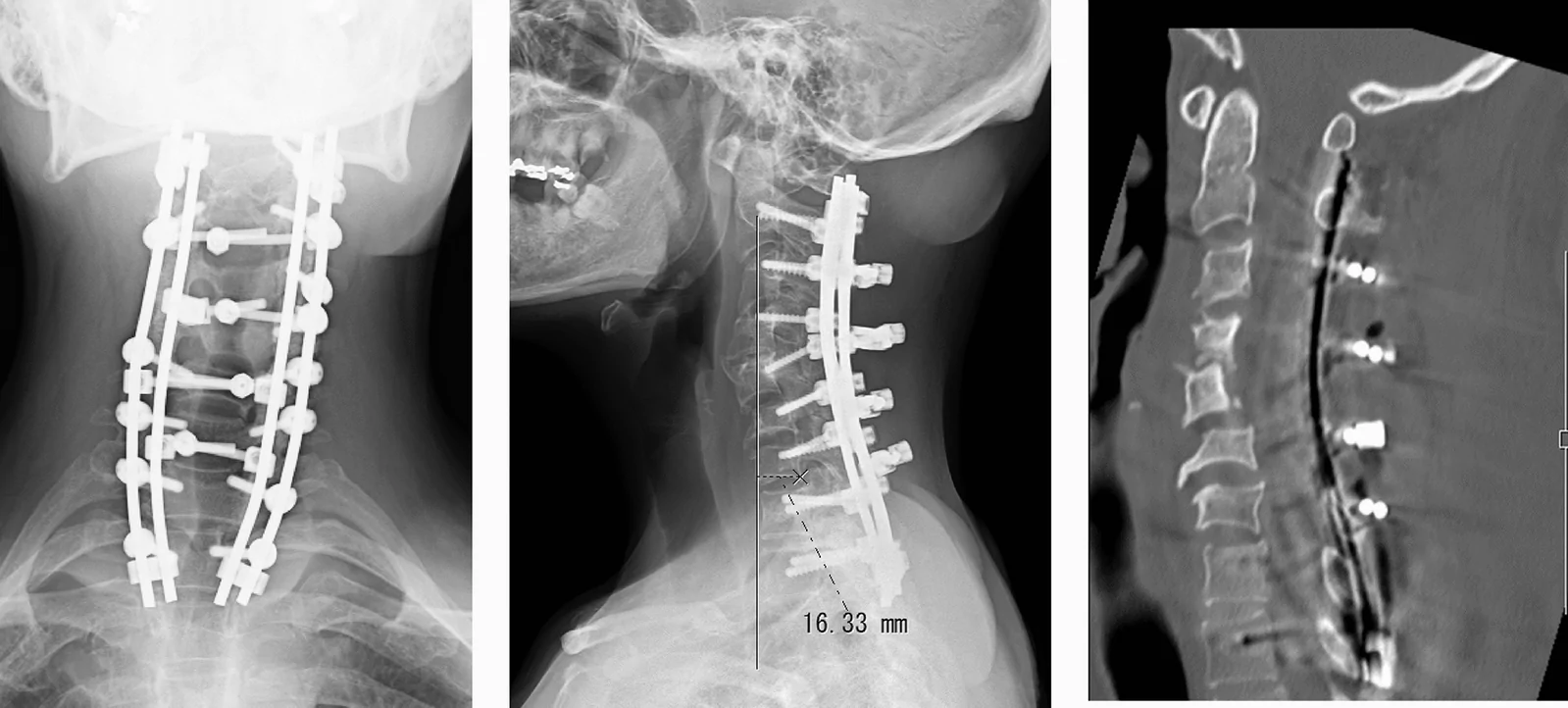
Postoperative X-rays and CT confirmed successful realignment of the C5 vertebral body

**Fig. 3:**
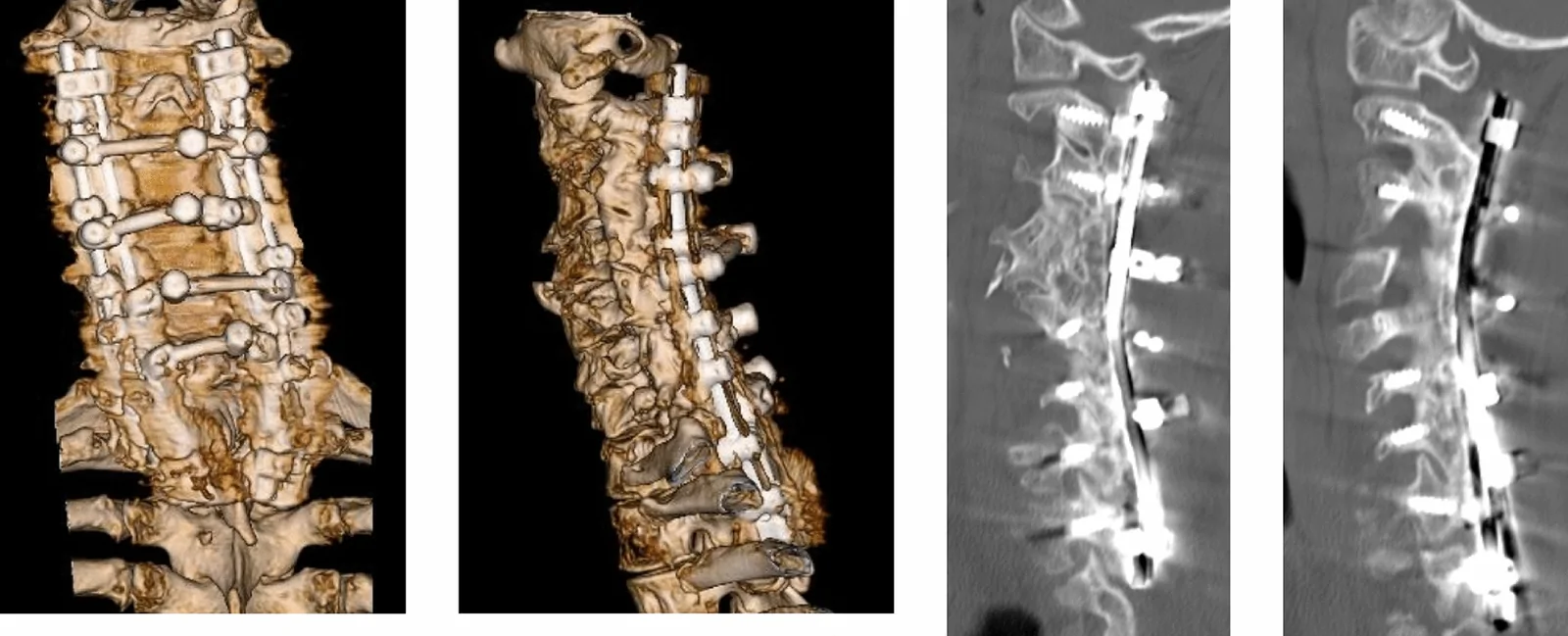
Successful bone fusion was confirmed by CT 2 years postoperatively

From a posterior approach, we performed laminectomy (C4-C6), fixation (C2-T2), and autologous bone grafting. For fusion, autologous iliac crest bone graft in addition to local bone graft was used. In cases of severe pedicle narrowing or dysplasia, lateral mass screws may be considered as an alternative to pedicle screws. In our patient, however, preoperative CT-based navigation planning enabled detailed assessment of pedicle morphology and trajectory simulation. As safe insertion was deemed feasible at all planned levels, pedicle screws were selected to achieve stable fixation. All screws were inserted as pedicle screw pathways with cobalt-chromium (CoCr) rods in addition to auxiliary rods for reinforcement. Following C4-C6 laminectomy, adequate spinal cord decompression was confirmed intraoperatively using motor evoked potential monitoring and ultrasonography, which demonstrated sufficient dorsal decompression without residual compression. No significant changes in neuromonitoring signals were observed during the procedure. Considering the pedicle narrowing and dystrophic anatomical changes associated with NF-1, all pedicle screws were inserted under CT-based navigation guidance to enhance accuracy. Screw placement was completed without major technical difficulty or implant-related complications. Intraoperative deformity correction was performed by contouring the rods to the intended sagittal alignment before fixation. The pre-bent rods were gradually connected to the pedicle screws to achieve controlled correction, thereby minimizing excessive mechanical stress on the screw anchors. Operative time was 538 min, and blood loss was 700 mL. No adverse procedure-related events were recorded. Immediately postoperative CT confirmed successful realignment of the C5 vertebral body (Fig. 2). His CL became 12°, and CSVA was 16 mm. Successful bone fusion was confirmed by X-rays and CT 2 years postoperatively (Fig. 3), with a JOA score improvement to 12.5 points. The JOA recovery rate was 52.6% both at 6 months and 2 years postoperatively. His preoperative CSVA of 26 mm indicated mild sagittal imbalance, which later improved to 16 mm, representing acceptable global sagittal alignment. Importantly, the focal dislocation at the C5/6 level was successfully reduced. Although the primary surgical goal was local deformity correction, his overall sagittal alignment was also improved to an acceptable range. We observed no visible correction loss for CL or CSVA. His visual analog scale pain score of 1 immediately after surgery had become 4 at 2 years postoperatively, which indicated slight worsening without the need for additional treatment or medication.

## Discussion

Reported cervical spine abnormalities in NF-1 have included dural sac and foraminal enlargement, scalloping of the vertebral bodies, pedicle narrowing, cervical deformity, vertebral subluxation, and spontaneous cervical dislocation, all of which often accompanied by severe cervical myelopathy [6–8]. To date, no universally recognized guidelines exist for surgical intervention in NF-1-related cervical deformity. Various factors are known to affect surgical approach selection, including kyphosis severity, range of deformed vertebrae, kyphotic curve flexibility, vertebra bone quality, location of neurologic compression, and the presence of intraspinal and/or paraspinal neurofibroma [9]. In the present case, we successfully employed posterior spinal fusion with the addition of accessory rods to manage progressive cervical deformity in NF-1.

There have been accounts of anterior only, posterior only, and combined anterior and posterior spinal fusion for NF-1 related kyphosis. However, the optimal choice remains controversial [10]. The previously reported surgical strategies and outcomes for NF-1-associated cervical deformity are summarized in Table 2. Posterior fixation surgery for NF-1-associated cervical deformities was found to harbor high incidences of pseudarthrosis and correction loss [3, 5]. Elsewhere, Ma et al. described instrumentation failure and kyphosis progression 4 years after surgery in a patient treated by an anterior approach, who ultimately required revision surgery [9, 11]. This approach is typically selected for skeletally mature patients with flexible localized moderate kyphosis (≤ 50°), limited range of fixation between C2-C7, and the absence of posterior neurologic compression. Indeed, the corrective ability of the anterior-only approach is limited by the insufficient pullout strength of the screws [9]. Lastly, Murlidharan et al. performed combined anterior and posterior surgeries on six patients. Some cases involved paraspinal neurofibromas, which led to significant bleeding and an average blood loss of 1,800 mL [12]. This approach is therefore considered highly invasive. Our posterior fusion method with accessory rods reduced bleeding compared with combined anterior–posterior surgery, offering a less invasive treatment option.

**Table 2:** Summary of surgical strategies for NF-1-associated cervical deformity

Author	Surgical strategy	Outcomes/complications
Parisini et al. [5]	Posterior-only fusion	High rate of pseudarthrosis and correction loss
Zhang et al. [10]	Posterior-only fusion with halo traction	Acceptable correction at ≥ 2 years follow-up
Ma et al. [11]	Anterior-only fusion	Instrumentation failure and kyphosis progression; revision required in some cases
Wang et al. [9]	Anterior-only fusion	Early and midterm outcomes acceptable; corrective power limited
Murlidharan et al. [12]	Combined anterior–posterior fusion	Strong stabilization with high invasiveness; mean blood loss > 1800 mL
Kakuta et al. [3]	Combined anterior–posterior fusion with vascularized fibula graft	Achieved fusion; complex reconstructive procedure
Present case	Posterior-only fusion with accessory rods	Achieved fusion at 2 years; no major complications

Regarding instrumentation materials, biomechanical studies have demonstrated greater rod stability for CoCr constructs than for titanium. CoCr rods may provide greater yield load and bending stiffness versus titanium rods, which can translate to better immediate stability during demanding corrections where robust fixation aids fusion [13]. Young’s modulus of titanium rods ranges from 105 to 117 GPa, while that of CoCr ranges from 230 to 240 GPa [14–17], supporting the notion that CoCr rods provide substantially greater stiffness. Biomechanically, increased rod stiffness may contribute to improved correction maintenance, particularly in cases of severe deformity or high mechanical demand. However, the clinical impact of this difference strongly depends on overall construct design and patient-specific factors. The use of accessory rods has been shown to enhance stability as well [18]. In recent years, we have implemented auxiliary rods for managing cases with a high risk of fixation failure, such as those with severe deformities caused by athetoid cerebral palsy or rheumatoid arthritis. In our reported patient, posterior fusion with accessory rods for severe NF-1-related cervical deformity achieved good bone union at 2 years postoperatively without any serious adverse events. A slight increase in VAS was observed at 2 years postoperatively. Possible causes include adjacent segment degeneration, implant-related discomfort, and muscular fatigue. However, no radiographic evidence of adjacent segment disease, implant loosening, or implant failure was identified. Although muscular factors may have contributed to the mild increase in pain, his symptoms did not require additional medical treatment, and no deteriorations in activities of daily living or overall functional status were noted.

While the use of accessory rods enhances overall construct rigidity, they are not segmentally coupled along all fixation points and may theoretically increase mechanical stress at the upper and lowest instrumented vertebrae. No junctional complications or implant-related problems were recorded during the follow-up period. However, increased stress at the terminal fixation points remains a potential concern; careful long-term follow-up is advised to monitor for possible proximal or distal junctional failure.

## Conclusions

We successfully managed a patient with severe NF-1-associated cervical deformity by posterior spinal fusion using a multi-rod construct. This method may be considered a viable option for progressive cases.

## Data Availability

Data will not be available.
